# Identification of Co-Expression Modules and Genes Associated With Tumor Progression in Oral Squamous Cell Carcinoma

**DOI:** 10.3389/pore.2022.1610481

**Published:** 2022-08-16

**Authors:** Zhijie Fang, Feifei Wang, Mengya Zhang, Hua Huang, Zhiqiang Lin

**Affiliations:** ^1^ Department of Otolaryngology, The Affiliated Suzhou Hospital of Nanjing Medical University, Suzhou Municipal Hospital, Gusu School, Nanjing Medical University, Suzhou, China; ^2^ Department of Nursing, Suzhou BenQ Medical Center, The Affiliated BenQ Hospital of Nanjing Medical University, Suzhou, China

**Keywords:** tumor progression, WGCNA, prognostic markers, OSCC, hub-gene

## Abstract

Oral squamous cell carcinoma (OSCC) is a common head-and-neck cancer with a deficiency of early diagnosis and poor prognosis. To identify potential diagnostic and prognostic markers of OSCC, we firstly used weighted gene co-expression network analysis (WGCNA) to build a co-expression module from GSE42743. Next, we performed Gene Ontology (GO) and Kyoto Encyclopedia of Genes and Genomes (KEGG) enrichment analyses on specified units from selected modules utilizing Database for Annotation, Visualization, and Integrated Discovery (DAVID). Additionally, we identified and validate hub genes of these specified modules from multiple datasets like GEPIA and TCGA. In total 16 co-expression modules were built by 17,238 genes of 74 tumor samples utilizing WGCNA. Through pathway and functional enrichment analysis, the turquoise module was most firmly relevant to the cell cycle, oocyte meiosis, and p53 signaling pathway. Hub genes VRK1, NUP37, HMMR, SPC25, and RUVBL1 were identified to be related to oral cancer at both molecular level and clinical levels. The expressions of these genes differed in tumor tissues and normal tissues. Meanwhile, patients with high hub gene expression had a poor prognosis clinically. To conclude, five hub genes were identified to be relevant to oral cancer from the molecular level and the clinical level. Therefore, the detection of these genes was of great significance. They can be regarded as diagnostic and prognostic biomarkers for oral cancer. Also, they could shed light on the improvement of patients’ overall survival and prognosis, which needs further analysis in the future.

## Introduction

Oral cancer ranks among the top 15 most common cancers in the world, characterized by delayed early diagnosis and a low five-year survival rate [1]. Oral cancer happens on the lip or oral cavity, and 90% of which is originated from squamous cells histologically. Tobacco [2], alcohol [3], betel quid [4] and human papillomavirus (HPV) [5] are important carcinogenic factors [6]. According to global cancer statistics 2018, the annual mortality rate of oral and lip cancer is about 177,384 and the annual number of new cases reached 354,864 [7]. Additionally, a recent statistical report has indicated that the incidence rate is higher in developed countries while the mortality is higher in developing countries [8]. The reasons for the low five-year survival rate can be divided into two main parts. Firstly, subtle precancerous lesions, and low prevalence of early screening result in delayed diagnosis. The disease has often developed to Stage III or IV when patients present for diagnosis [9, 10]. On the other hand, because of the lack of specific biomarkers, there is no specific curative treatment for oral cancer. Under the influence of a low early diagnosis rate and nonspecific treatment, the five-year survival rate for patients with oral cancer has not ameliorated, and has been stuck at 50%–55% for the past several decades [1].

Weighted gene co-expression network analysis (WGCNA) has been applied to effectively detect highly correlated gene clusters, which can be used as a gene screening tool [11]. DAVID and other databases are used to promote the analysis of genome-scale datasets. The use of these genetic analysis tools and multiple databases is conducive to the accurate screening of genes, to identify specific biomarkers and prognosis-related genes of oral cancer.

## Materials and Methods

### Data Procession

The OSCC dataset of GSE42743 was obtained from NCBI Gene Expression Omnibus (GEO) (https://www.ncbi.nlm.nih.gov/geo/). The GSE42743 was an expression profiling based on GPL570 platform (Affymetrix Human Genome U133 Plus 2.0 Array) and included 103 samples consisting of 24 matched normal and tumor samples from the same patient [12]. The R package affy (under the R environment, version 3.6.1) was used to preprocess the gene expression profiles. After RMA normalization and probe annotation. We selected 74 tumor samples from the dataset with 17238 genes, the top 50% of genes with the greatest variance changes are used to further establish the co-expression network. After validation, the gene expression array was constructed into a gene co-expression network using the R package WGCNA. First, the adjacency matrixαij is constructed by the following formula to calculate the connection strength between nodes:

αij=|(1+cor(xi+yi))/2|β

where xi and xj were the expression levels of the gene i and the gene j respectively. β stands for soft threshold. A suitable soft threshold can ensure that the co-expression network conforms to the scale-free network.

The next step is to convert the adjacency matrix into a topological matrix, TOM (topological overlap measure) is used to describe the degree of association between genes. The formula is as follows:

TOM=(∑μ≠ijαiμαμj+αij)/(min(∑μαiμ+∑μαjμ)+1−αij)

Based on TOM difference, the genes with similar expression patterns are classified into the same module by hierarchical clustering function, and the minimum size of the gene tree diagram was 30 genes. A Dynamic Tree Cut algorithm was used to classify the genes and visualize the network. Finally, the gene network of characteristic genes was visualized.

### Identification of Clinical Significant Modules

The most representative gene in each module is called the module eigengenes (MEs), which represents the overall level of gene expression in the module and is the first principal component in each module. In order to identify the clinical significant module, we calculated the correlation between MEs and clinical phenotypes. Gene significance (GS) was used to measure the correlation between each gene and external information. It is defined as the log10 transformation of the *p* values (GS = lgP) in linear regression results of gene expression and clinical phenotype. Moreover, module significance (MS) was defined as the mean GS of all genes in a certain module. The module with the highest MS is considered the most relevant to the clinical trait.

### Function Enrichment Analysis

To learn more about the biological functions of genes in key modules. We used an online database for annotation, visualization, and integrated discovery (DAVID, http://david.abcc.ncifcrf.gov/) to accomplish Gene Ontology (GO). The gene list of the key module was uploaded, and we found the result of the biological process (BP) and KEGG pathway. A *p*-value ≤0.05 was considered significant.

### Hub Gene Identification and Validation

Gene connectivity was measured by the absolute value of the Pearson correlation. Genes with high intra-module connectivity were considered the central genes of the module and may have important biological functions. Genes with correlation >0.8 were selected for further verification. GEPIA (http://gepia.cancer-pku.cn/) is a newly developed interactive web server for analyzing the RNA sequencing expression data of 9,736 tumors and 8,587 normal samples from the TCGA and the GTEx projects, using a standard processing pipeline [13]. It provides the patient survival analysis of candidate genes, and the significant results were picked out (log-rank *p* ≤ 0.05). In order to further verify the selected genes, we downloaded the OSCC mRNA sequencing data from The Cancer Genome Atlas Project database (TCGA, https://cancergenome.nih.gov/) and standardized the data by TPM. The Human Protein Atlas (http://www.proteinatlas.org) was also used to verify the expression of selected genes at the translation level.

### Pathway Enrichment Analysis

For further understanding of the pathway gene sets related to the hub genes, Gene set enrichment analysis (GSEA) and GSVA analysis were performed. GSEA was performed using GSEA v4.1.0 software (http://www.gsea-msigdb.org/gsea/) [14, 15] and the gene sets background was c2.cp.kegg.v7.2.symbols.gmt. GSVA was conducted with the GSVA package [16]. The samples were divided into two groups according to the median expression of hub genes and R package Limma was used to calculate the difference.

### GSCALite

The relation between the small molecule/drug sensitivity (IC50) and the hub genes expression profile in HNSC cell lines in CTRP was analyzed utilizing Webtool GSCALite (http://bioinfo.life.hust.edu.cn/web/GSCALite/). Calculation of correlation was performed by Spearman’s correlation analysis. Moreover, the hub genes methylation level was also identified in HNSC using GSCALite, the t test was performed to define differences in methylation between tumor and normal samples.

### TIMER

We analyze the relation between the abundance of tumor immune infiltrating cells and hub genes by TIMER (Tumor Immune Estimation Resource) (https://cistrome.shinyapps.io/timer/). Correlation analysis was performed using Spearman’s correlation test.

## Result

### Data Collection and Sample Cluster

**FIGURE 1:**
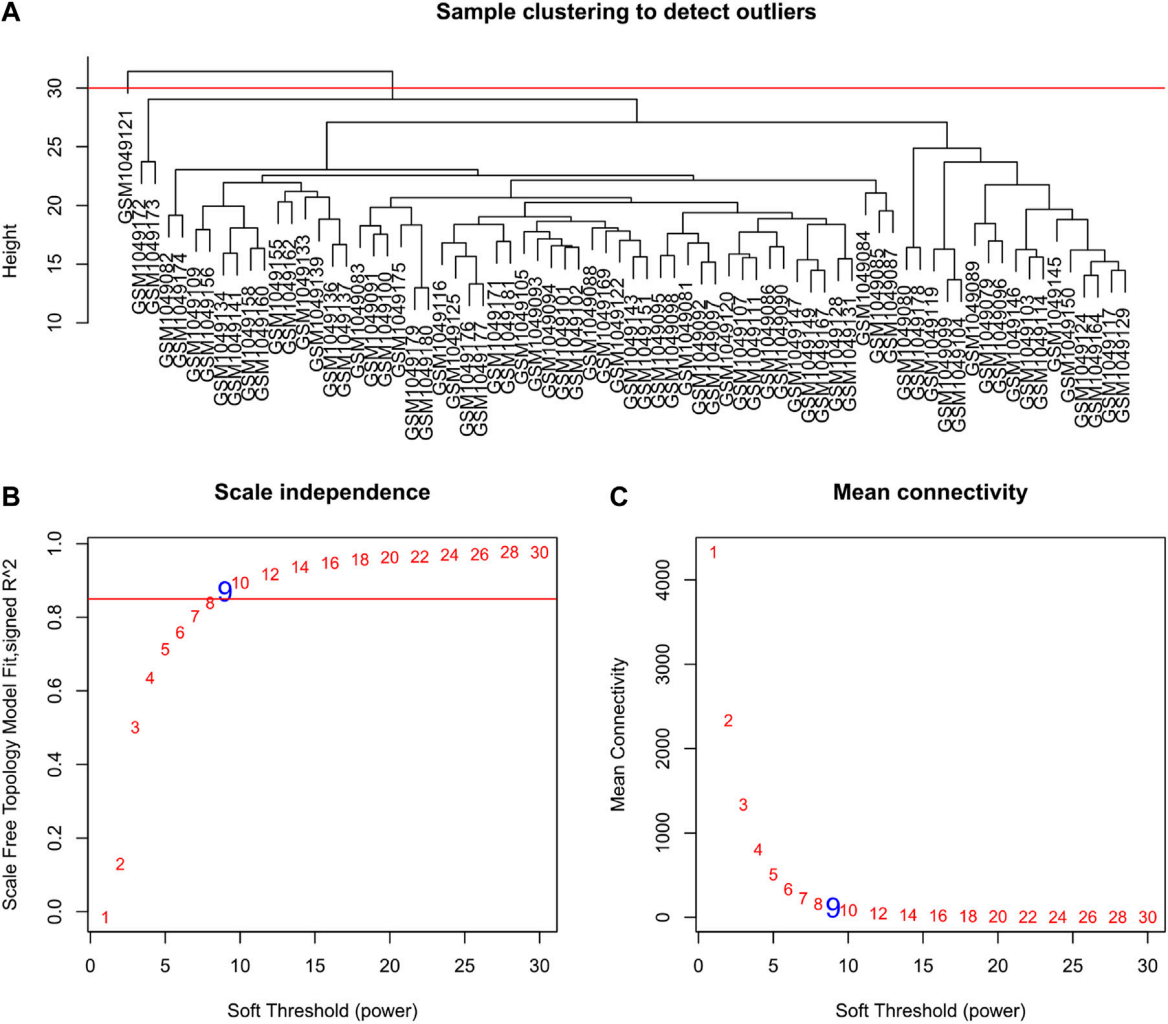
Clustering dendrogram of samples and set of soft-thresholding power. **(A)** Sample clustering was conducted to detect outliers. The outliers GSM1049121 were removed from this study; **(B,C)** Verification of selected soft threshold power. Analysis of scale independence and mean connectivity was performed to attain the suitable for the scale-free topology.

**FIGURE 2:**
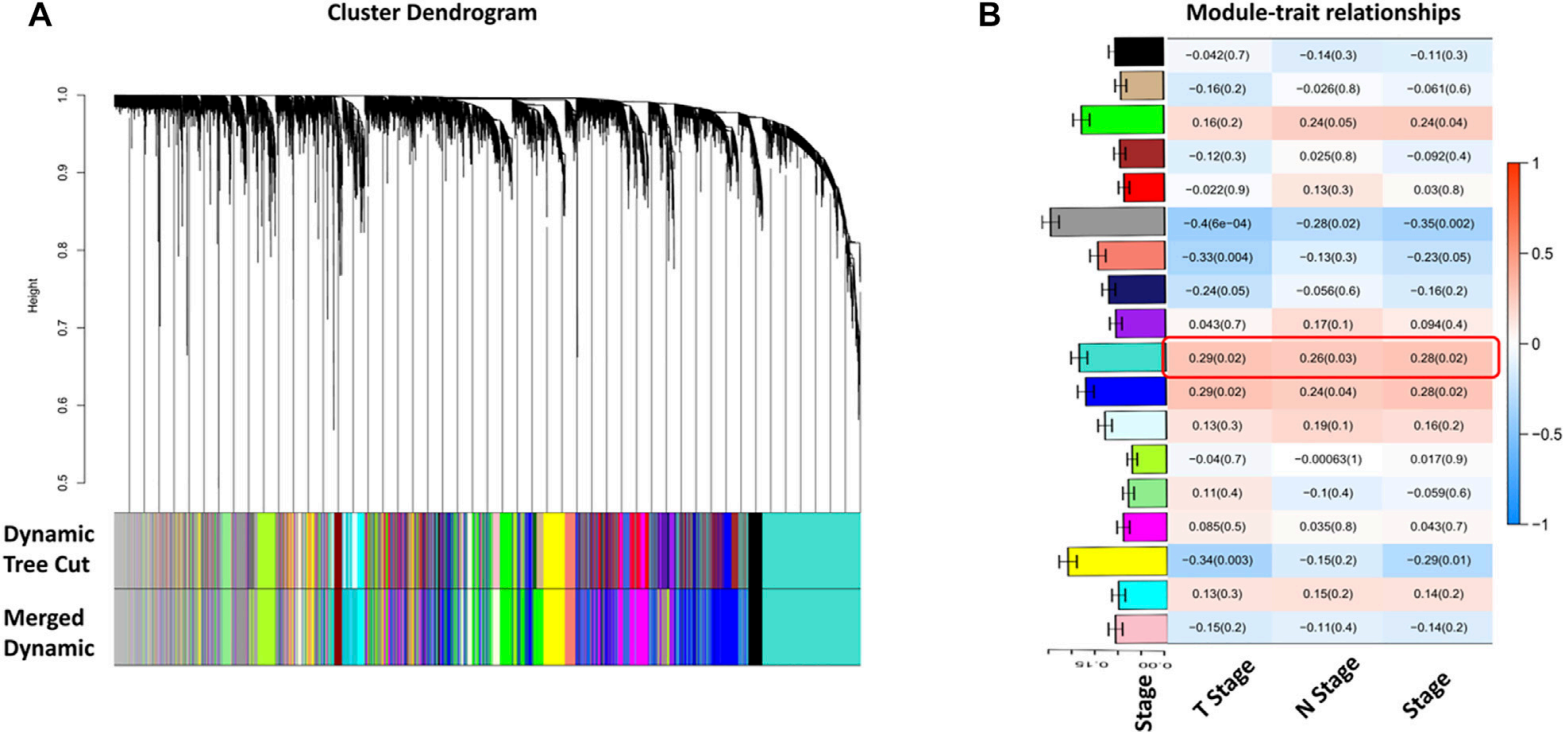
Identification of tumor-related gene modules. **(A)** Cluster dendrogram obtained by hierarchical cluster analysis. Each module was corresponding assigned to one color; **(B)** Heatmap with barplot of correlation between modules and clinical traits of HNSC. The red color block represented positive correlation, and the blue block represents negative correlation. The number above in the cell represents Spearman’s correlation and the number in brackets represents the *p*-value. The black module was most related to the tumor progression. The barplot on Y-axis indicated the average gene significance with the TNM stage.

Selecting tumor samples in GSE42743, the data had a total of 74 samples and three phenotypes. Subsequently, cluster analysis was performed on the samples and the outlier GSM1049121 had been removed, then clustered the samples with phenotypic information using Pearson correlation (Figure 1). In order to ensure that the established co-expression network conformed to the scale-free network, we determined the soft threshold as β = 9 and verified the average connectivity and correlation coefficient (R2) of the selected soft threshold (Figure 1), R2>0.9 indicated that the selected β value could build a gene scale-free network. We used average-linkage hierarchical clustering to cluster genes and set the MEDissThres parameter to 0.25 to merge similar modules. Finally, 16 gene modules were obtained, of which the turquoise module was the most relevant to the tumor process (Figure 2).

### Gene Ontology and Pathway Enrichment Analysis

**FIGURE 3:**
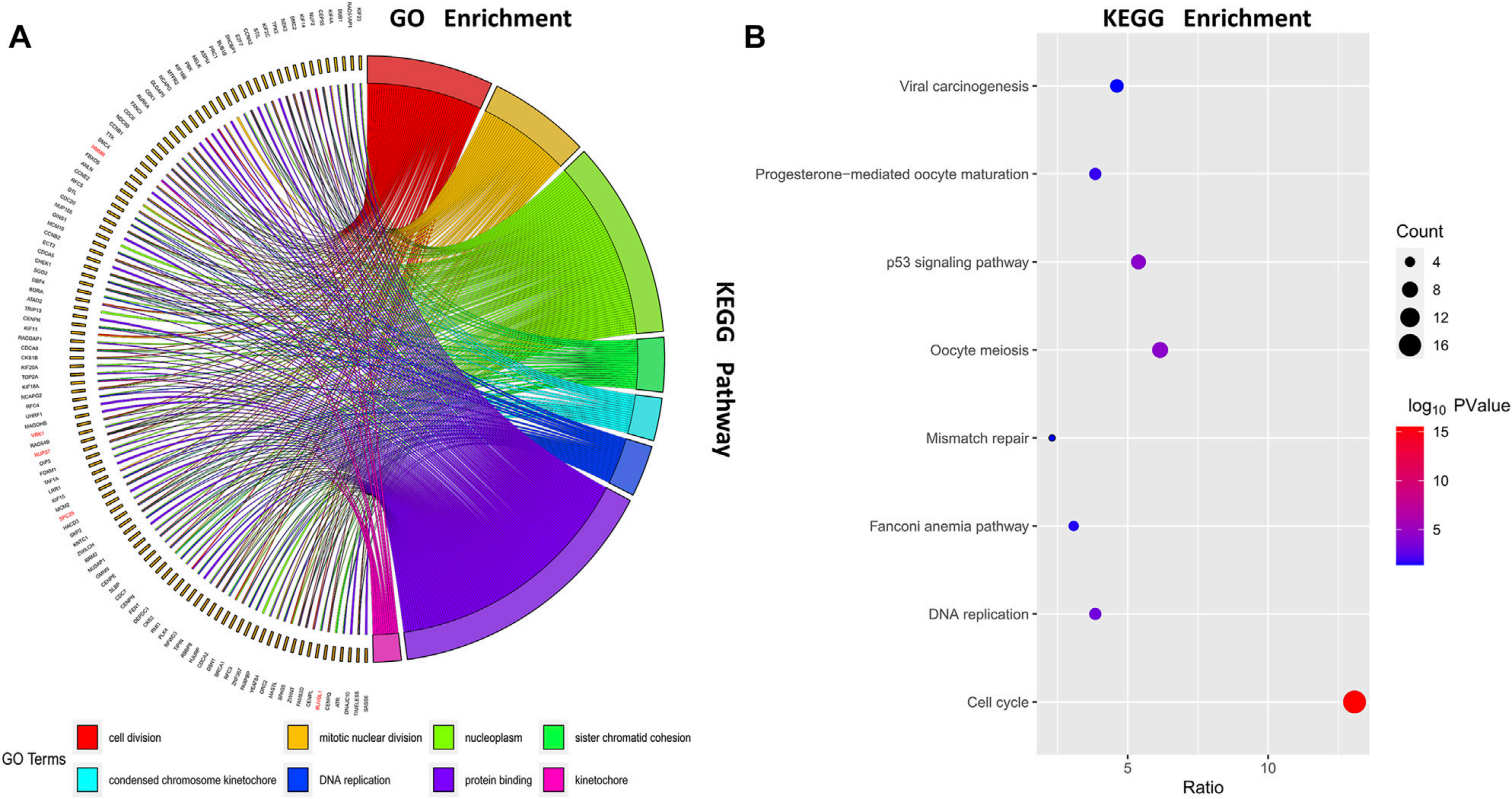
Function enrichment analysis. Gene ontology (GO) enrichment analysis and Kyoto Encyclopedia of Genes and Genomes (KEGG) enrichment analysis were conducted to identify significant biological processes; **(A)** Chord diagram of GO enrichment; **(B)** Bubble diagram of KEGG enrichment.

We selected genes in the turquoise module and uploaded them to DAVID for functional enrichment analysis. Select the BP function group in Gene ontology, the 8 most significant biological processes are shown in Figure 3A. The results show that the turquoise modules were mainly enriched in the process of cell division such as cell division, mitotic nuclear division, and sister chromatid cohesion. We also performed a KEGG pathway analysis on the turquoise module to investigate possible pathways, pathways with *p* values > 0.05 are shown in Figure 3B. As can be seen from the results, the turquoise modules were mainly enriched in the cell cycle-related pathways such as cell cycle, oocyte meiosis, and p53 signaling pathway.

### Identification and Validation of Hub Genes

**FIGURE 4:**
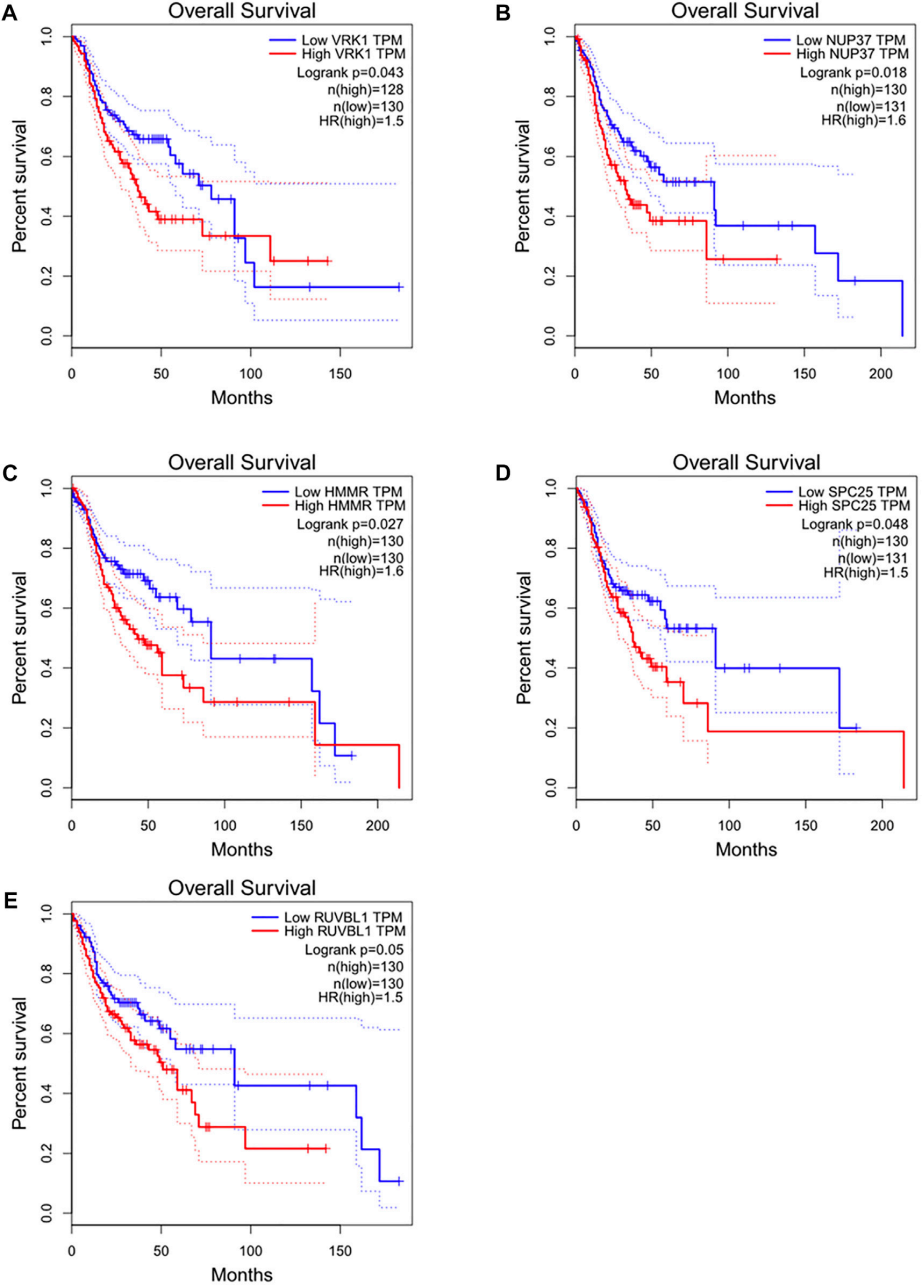
Overall survival and survival analysis of hub genes in HNSC. Kaplan–Meier analysis showed that patients with higher expression levels of hub genes exhibited worse OS. **(A)** VRK1, **(B)** NUP37, **(C)** HMMR, **(D)** SPC25, **(E)** RUVBL1, the order of pictures below is the same.

**FIGURE 5:**
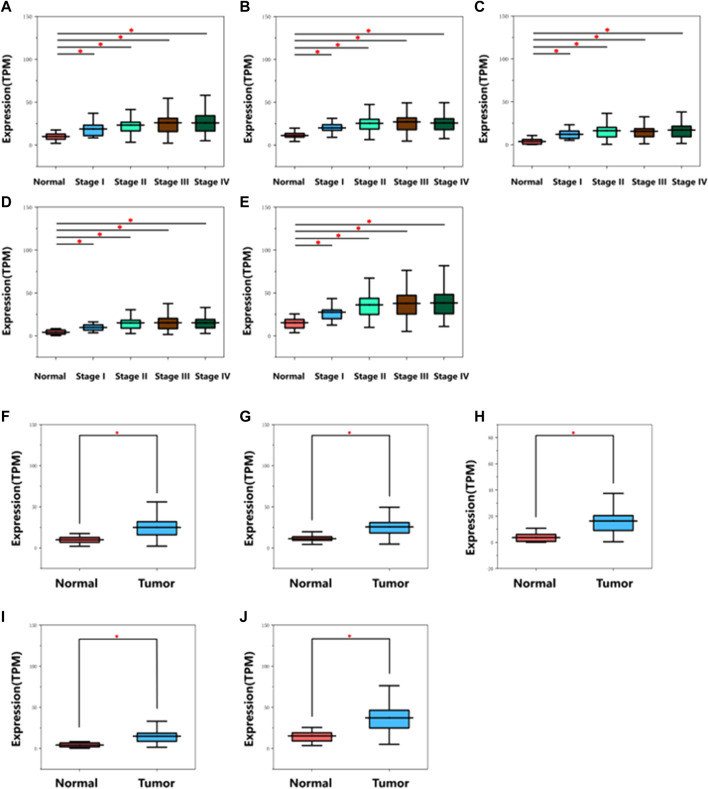
Diagram of mRNA expression level of hub genes in different stages on TCGA database. **(A)** VRK1, **(B)** NUP37, **(C)** HMMR, **(D)** SPC25, **(E)** RUVBL1. Diagram of mRNA expression level of hub genes in normal and tumor tissues on TCGA database. **(F)** VRK1, **(G)** NUP37, **(H)** HMMR, **(I)** SPC25, **(J)** RUVBL1. *indicates *p* < 0.05. The gene expression was remarkably up-regulated in cancer tissues.

Detailed gene lists of each module were provided in Supplementary Table S1. The 94 genes with a correlation >0.8 in the turquoise module were the hub genes to be verified. We performed survival analysis on the GEPIA, and results of *p* < 0.05 were considered statistically significant. Genes with significant survival analysis results were considered to be the hub genes. Figure 4 shows survival analysis results of hub genes. They were VRK1, NUP37, HMMR, SPC25, and RUVBL1 and patients with high expression had lower overall survival. Furthermore, TCGA data showed the expression of these five genes in tumor tissues was significantly higher than that in normal tissues, and they were up-regulated in advanced tumor tissues (Figure 5). In addition, based on The Human Protein Atlas (HPA), the protein expression levels of these five genes were significantly higher in tumor tissues than in normal tissues (Supplementary Figure S1).

### Pathway Enrichment Analysis

**FIGURE 6:**
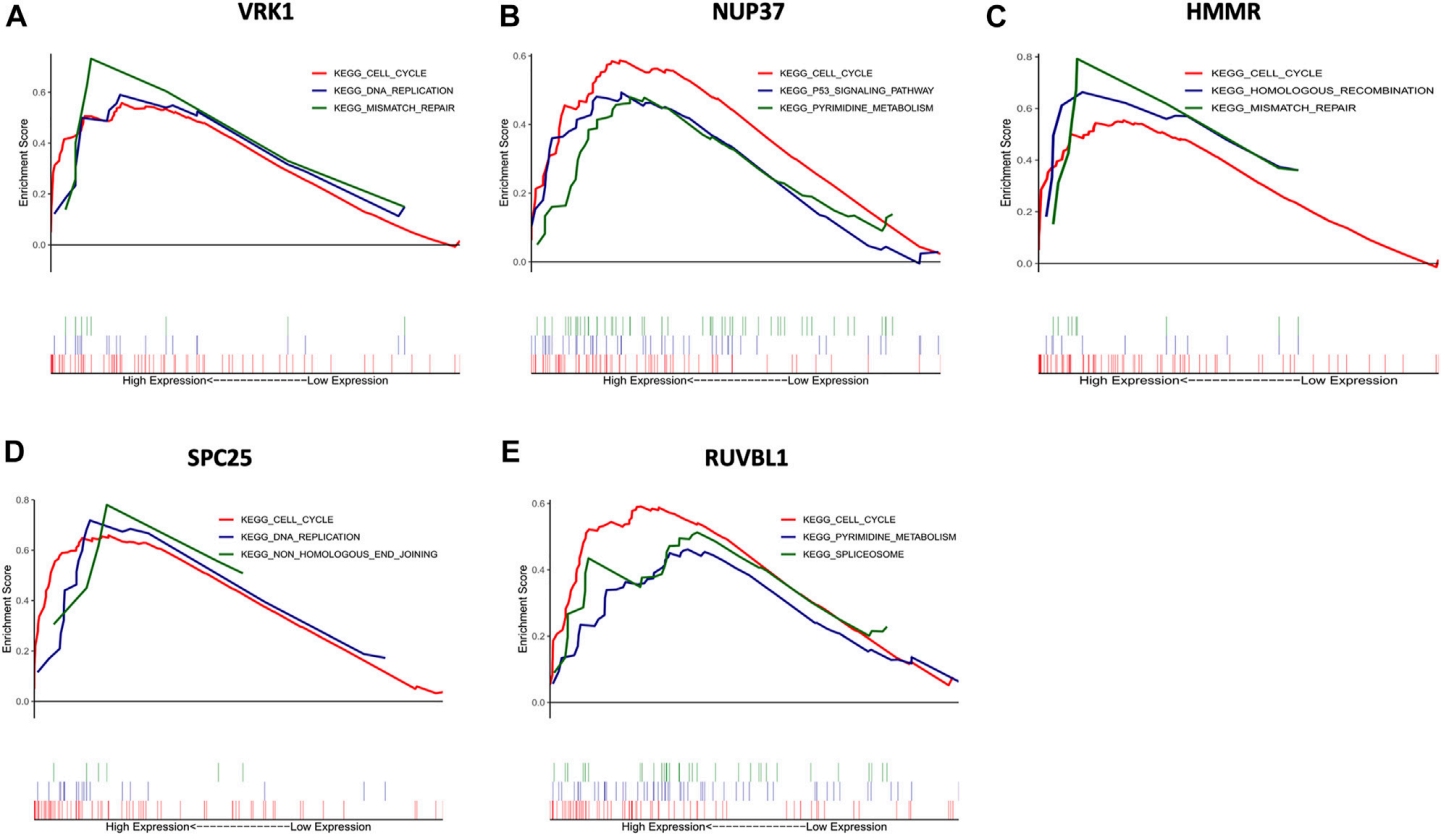
Gene set enrichment analysis (GSEA) of hub genes. The top three functional gene sets enriched in HNSC were listed. **(A)** The top three pathways are enriched in high VRK1 patients. **(B)** The top three pathways are enriched in high NUP37 patients. **(C)** The top three pathways are enriched in high HMMR patients. **(D)** The top three pathways are enriched in high SPC25 patients. **(E)** The top three pathways are enriched in high RUVBL1 patients.

To further insight into the potential biological functions of hub genes, GSEA and GSVA were performed for pathway enrichment. Figure 6 shows the top three up-regulated pathways of each hub gene (based on the *p*-value), all hub genes were enriched in the cell cycle. Furthermore, HMMR and VRK1 were enriched in mismatch repair. SPC25 and VRK1 were enriched in DNA replication. NUP37 was enriched in the P53 signaling pathway.

**FIGURE 7:**
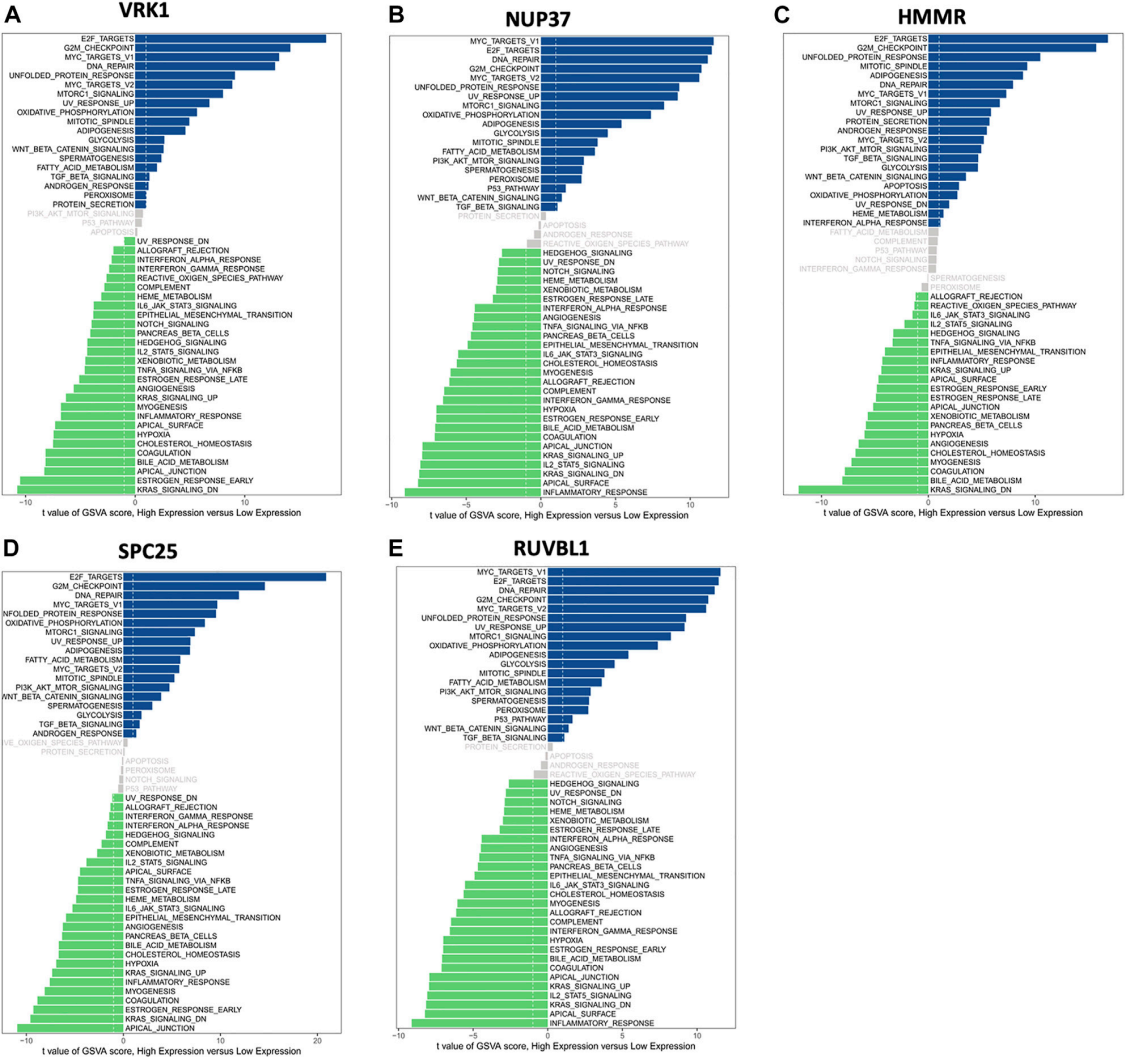
Gene set variation analysis (GSVA) of hub genes showed differentially expressed pathways of each hub gene. **(A)** The differential pathways between high VRK1 and low VRK1 patients. **(B)** The differential pathways between high NUP37 and low NUP37 patients. **(C)** The differential pathways between high HMMR and low HMMR patients. **(D)** The differential pathways between high SPC25 and low SPC25 patients. **(E)** The differential pathways between high RUVBL1 and low RUVBL1 patients.

Moreover, GSVA was used to investigate the potential biological process and signaling pathways. Figure 7 shows that high HMMR, SPC25, and VRK1 expressions were enriched in E2F targets and G2M checkpoint pathways. As for NUP37 and RUVBL1, the first two enriched pathways for high expression groups are MYC targets V1 and E2F targets. For low expression groups, KARS signaling DN’s enriched most in HMMR and VRK1, and apical junction enriched most in SPC25. Low NUP37 and RUVBL1 expression was enriched in inflammatory response.

### GSCALite

As is shown in the bubble heatmap (Supplementary Figure S2), the expression of VRK1 was negatively correlated with IC50 of most drugs in OSCC, indicating that VRK1 is sensitive to most drugs and can be used as a potential therapeutic target. RUVBL1 is negatively correlated with IC50 of half of the drugs and can also be used as a reliable therapeutic target. Moreover, SPC25 only shows negatively correlated with neopeltolide and methotrexate. Most notably, methotrexate is sensitive to all three hub genes (VRK1, RUVBL1, and SPC25). However, NUP37 may be resistant to BRD−K30019337. Hub genes’ response to drugs has great value for targeted drug design and clinical therapy. Supplementary Figure S3 shows the methylation level of five hub genes. In detail, VRK1 and SPC25 were significantly lower in the tumor sample, but differences in NUP37, RUVBL1, and HMMR did not reach statistical significance.

### TIMER

**FIGURE 8:**
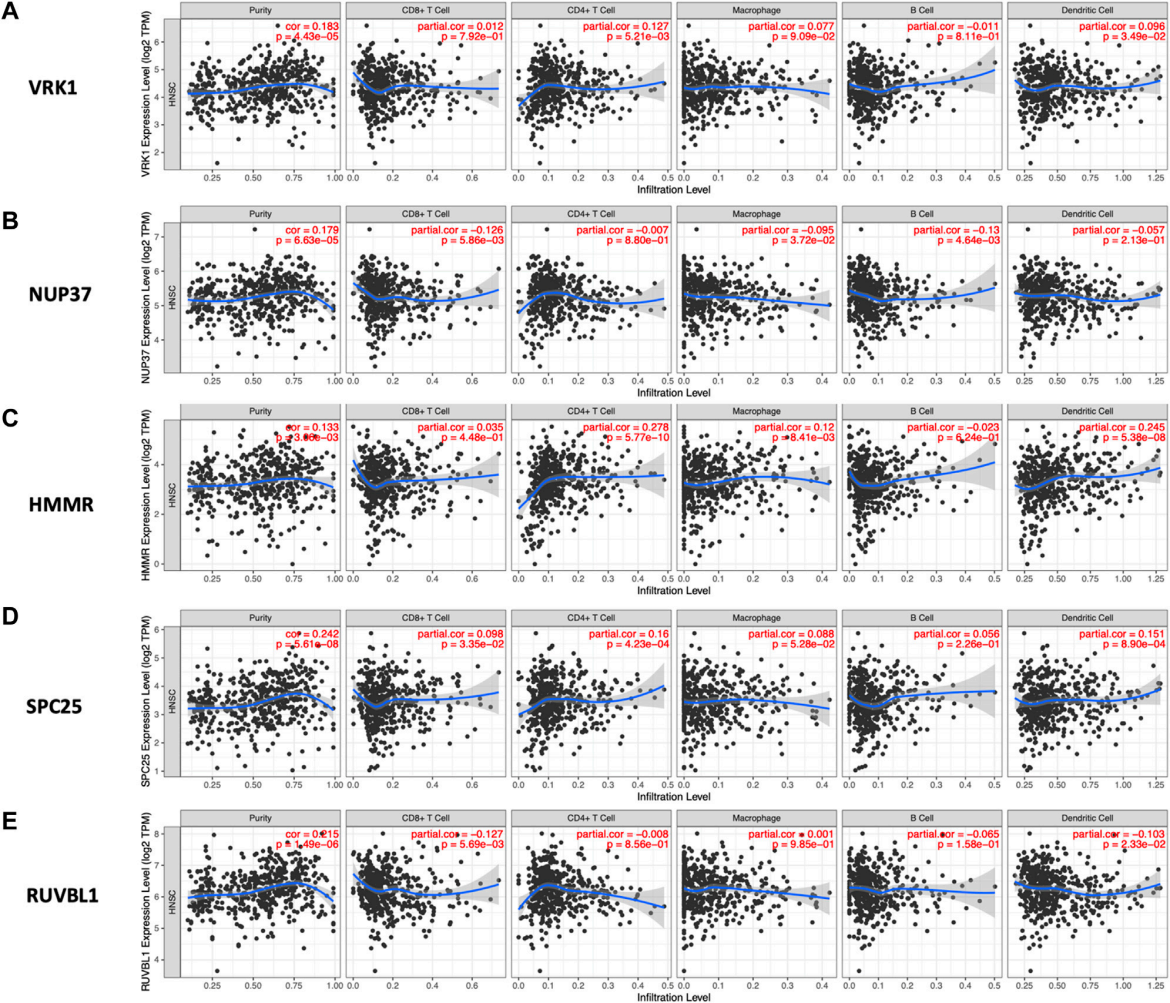
Relationship between the expression level of hub genes and infiltration level of various immune cells analyzed by TIMER. The spearman’s correlation between the expression level of **(A)** VRK1, **(B)** NUP37, **(C)** HMMR, **(D)** SPC25, and **(E)** RUVBL1 and five immune cells (CD4^+^ T Cell, CD8^+^ T cell, Macrophage, B cell, and Dendritic Cell).

As is shown in Figure 8, the high expression of HMMR, SPC25, and VRK1 was correlated with increased infiltration of CD4+cells and dendritic cells. The infiltration level of CD8+cells was negatively associated with NUP37 and RUVBL1. Notably, NUP37 and RUVBL1 also exhibited the downregulated tendency in increased infiltration of all immune cells.

## Discussion

Because of the low examination rate and poor diagnosis rate in the early stages of OSCC, the prognosis of oral cancer has not improved with the progress of treatment. Thus, identifying effective biomarkers correlating with the development of OSCC can greatly help in the screening strategies and targeted therapy for OSCC. In the present study, the mRNA expression data were downloaded from GEO, and a co-expression network was constructed through WGCNA. Subsequently, the turquoise module was identified to be most significantly associated with the OSCC stages and smoking. Function enrichment analysis of turquoise module was performed by DAVID database, and validation of the hub genes in the turquoise module was performed based on TCGA database at the transcriptional level. Moreover, by performing survival analyses, it was demonstrated that oral cancer patients with high expression levels of hub genes have a poor prognosis utilizing GEPIA. More convincingly, immunohistochemistry also verified the results based on HPA. In conclusion, we finally identified five genes most associated with tumor progression and prognosis: VRK1, NUP37, HMMR, SPC25, and RUVBL1.

Lohavanichbutr et al reported a 13 genes signature in HPV-negative OSCC patients in 2013 and confirmed that the 13 genes signature showed better accuracy in predicting prognosis than the TNM stage through ROC analysis [12]. However, the potential of the signature of the 13 genes has not been explored from the aspects of biological function, immune infiltration, and drug sensitivity. Considering the convenience of clinical application, our research focuses on finding a single gene biomarker to assist in the diagnosis of OSCC patients. We confirmed the prognostic efficacy of five hub genes in the external dataset TCGA and tried to validate the expression of hub genes at the protein level through the HPA database. Moreover, we also explored the biological function, immune infiltration, and small molecule drug sensitivity of hub genes through the GO, GSEA, GSVA analysis, and online database: TIMMR and GSVALite. We believe that our study is a valuable reanalysis of the data provided by Lohavanichbutr et al., and is expected to provide new insights into the diagnosis and targeted treatment of OSCC patients.

Pathway enrichment analysis indicated that these hub genes may be involved in the cancer-associated signal pathway, like p53 and KARS signaling DN’s to prove tumor progression. Abnormal DNA methylation is one of the epigenetic changes associated with gene silencing, while normal somatic cells are generally unmethylated [17]. Hence, the result from GSCALite showed that VRK1 and SPC25 were not susceptible to epigenetic silencing, leading to a bad prognosis for patients. TIMER revealed the correlation between hub genes and the abundance of tumor immune infiltrating cells. High expression of hub genes related to increased immune cell infiltration suggested the presentation of tumor antigen and immune response. On the contrary, decreased tendency of immune infiltration may suppress host immune responses and lead to a worse prognosis [18].

Vaccinia-related kinase 1 (VRK1), is a member of the VRK family of serine/threonine kinases. It controls the early process of the cell cycle and influences different cell cycle phases according to protein level and activation degree [19]. The higher level of VRK1 in the S phase indicates that VRK1 plays an important role in promoting DNA replication [20]. As for expression in tumors, VRK1 has been detected to be highly expressed in a variety of cancers, such as head and neck squamous cell carcinomas (HNSCC), and lung cancers especially with p53 mutations[21, 22]. Namgyu Lee et al reported that higher levels of VRK1 can suggest poorer prognosis, shorter overall, and higher recurrence rates [23]. Another study on the expression of VRK1 in breast cancer showed the same conclusion [24]. VRK1 is a widely-detected gene, and its relationship with the cell cycle and cancer progression is relatively clear, which is helpful for the accuracy of our verification.

NUP37 is a component of the nuclear pore complex (NPC). It is found to be both significantly mutated genes (SMGs) and tumor-specific disruptive genes (TDGs) in OSCC specimens, but its role in the process of OSCC has not been clarified in this study [25]. In other cancers, it was reported to be remarkably up-regulated in Hepatocellular carcinoma (HCC) which can promote the growth, migration, and invasion of HCC through activating YAP/TEAD signaling [26]. A recent report showed that the expression of NUP37 in advanced NSCLC was significantly higher than that in early NSCLC, and the high expression of NUP37 indicated poor overall survival [27]. These findings are consistent with our results, which reveal the role of NUP37 in promoting tumor progression and affecting prognosis.

Hyaluronan-mediated motility receptor (HMMR) is highly related to the tumor process because of hyaluronan-mediated signaling. HMMR regulates spindle assembly in mitotic cells, so an elevated expression of HMMR can be detected in actively proliferative tissues, like neoplastic tissues [28]. However, in cancer, HMMR is not only related to tumor progression, but also to tumor invasion, metastasis, and prognosis. Kiran et al reported that HMMR was involved in the pathogenesis of malignant peripheral nerve sheath tumor (MPNST) [29]. Li et al found that the detection rates of HMMR increased with the tumor process of gastric cancer by pathological and immunohistochemical examination of different stages of gastric cancer specimens [30]. Assmann et al found that higher expression of HMMR elevated movability and invasiveness of breast cancer cells [31]. HMMR is rarely reported in squamous cell carcinoma, so our novel findings need further investigation.

Spindle polar component 25 (SPC25), a component of Ndc80 complex, controls spindle assembly checkpoints in mitosis [32]. Reports showed that elevated expression of SPC25 can increase cancer stem cell (CSC) properties and predict poor prognosis. In non-small cell lung adenocarcinoma, SPC25 knockout reduced CSC characteristics and invasiveness of A549 cells. And in lung adenocarcinoma, SPC25 was identified to be an independent prognostic factor for a worse survival rate [32]. In prostate cancer (PrC), Cui et al found SPC25 + PrC produced more tumor spheres, which also had stronger resistance to chemotherapeutic drugs-induced cell apoptosis compared with SPC25- PrC. Their results showed that SPC25 can regulate the stemness of prostate cancer cells [33]. Studies of breast cancer and liver cancer research also reached the same conclusion. SPC25 was significantly up-regulated in tumor tissue, and can independently predict the prognosis of patients [32, 34].

RUVBL1 is a highly conserved AAA+ ATPase in eukaryotic cells and participates in tumor progression [35]. Many specific RUVBL1-involved signaling pathways have come to light, which means that RUVBL1 has been confirmed as a tumor therapeutic target. Guo et al have found that RUVBL1 can inhibit the phosphorylation of c-raf protein at serine 259, thus activating the Raf/MEK/ERK pathway and promoting tumor progression [35]. Yuan et al reported that in lung adenocarcinoma cells, RUVBL1 knockdown caused arrested G1/S phase cell cycle and decreased proliferation of A549 and h292 cells due to repression of the AKT/GSK-3β/cyclin D1 pathway [36]. These pathways provide a good reference for the specific mechanism of RUVBL1 on OSCC.

WGCNA can identify the relationship between gene expression patterns and clinical traits in an unsupervised manner, which makes the results have significant biological implications. However, we acknowledge several potential limitations of this study. Firstly, like most other statistical analyses, the results of WGCNA may be biased or invalid when tissues are contaminated. Additionally, because of insufficient funding, the results have not been validated by experiments. Although hub genes have good prognostic value in OSCC in general, tumors at different localizations, TNM stages, and ages may have different clinical outcomes, which requires further subgroup analysis. Further clinical experiments are required to better substantiate the findings of this study. Last, due to the limited sample size, it is necessary to verify the results using more datasets.

In conclusion, we identified five hub genes (VRK1, NUP37, HMMR, SPC25, RUVBL1) associated with oral carcinogenesis and progression, which may serve as effective prognostic indicators for OSCC and potential therapeutic targets in OSCC treatment.

## Data Availability

The original contributions presented in the study are included in the article/Supplementary Material, further inquiries can be directed to the corresponding author.

## Supplementary Material

Supplementary Figure S1 Immunohistochemistry of hub genes based on the Human Protein Atlas. Immunohistochemistry of the five hub genes based on the Human Protein Atlas. **(A)** Protein levels of VRK1 in normal tissue (staining: low; intensity: weak; quantity: >75%). **(B)** Protein levels of VRK1 in tumor tissue (staining: medium; intensity: moderate; quantity: 25–75%). **(C)** Protein levels of NUP37 in normal tissue (staining: not detected; intensity: weak; quantity: <25%). **(D)** Protein levels of NUP37 in tumor tissue (staining: medium; intensity: moderate; quantity: >75%). **(E)** Protein levels of HMMR in normal tissue (staining: l not detected; intensity: negative; quantity: none). **(F)** Protein levels of HMMR in tumor tissue (staining: low; intensity: moderate; quantity: <25%). **(G)** Proteins level of RUVBL1 in normal tissue (staining: not detected; intensity: weak; quantity: <25%). **(H)** Protein levels of RUVBL1 in tumor tissue (staining: low; intensity: weak; quantity: <25%). **(I)** Protein levels of SPC25 in normal tissue (staining: not detected; intensity: negative; quantity: none). **(J)** Protein levels of SPC25 in tumor tissue (staining: medium; intensity: moderate; quantity: 25%–75%).

Supplementary Figure S2 Drug sensitivity analysis of hub genes based on GSCALite. The spearman’s correlation between the expression of each hub gene and the IC50 of each drug was shown. The blue spot represents drug sensitivity, and the red spot represents drug resistance.

Supplementary Figure S3 Difference of methylation level of hub genes in tumor and normal tissue based on GSCALite. Blue represents a negative correlation between the expression of hub genes and the methylation level of hub genes.

Supplementary Table S1 The detailed list of module genes.
