# Supplementary figures and images for: Identification of Co-Expression Modules and Genes Associated With Tumor Progression in Oral Squamous Cell Carcinoma
Source: Pathol Oncol Res. 2022 Aug 16;28:1610481. doi: 10.3389/pore.2022.1610481 (PMC9426548; doi:10.3389/pore.2022.1610481)

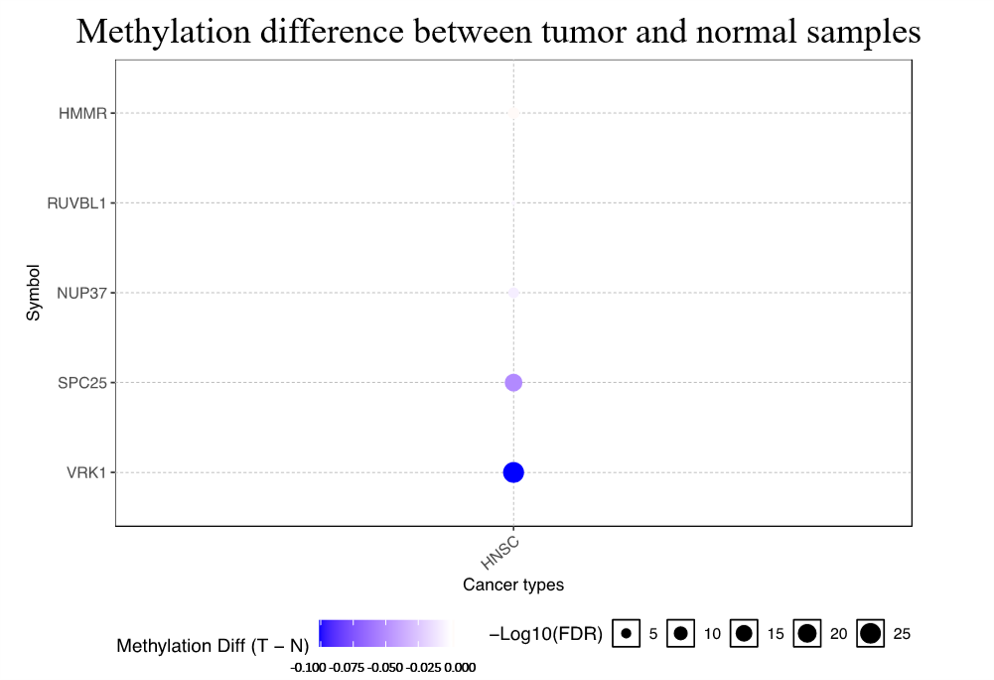

Supplement: Supplementary file 1 [file Image3.tif]

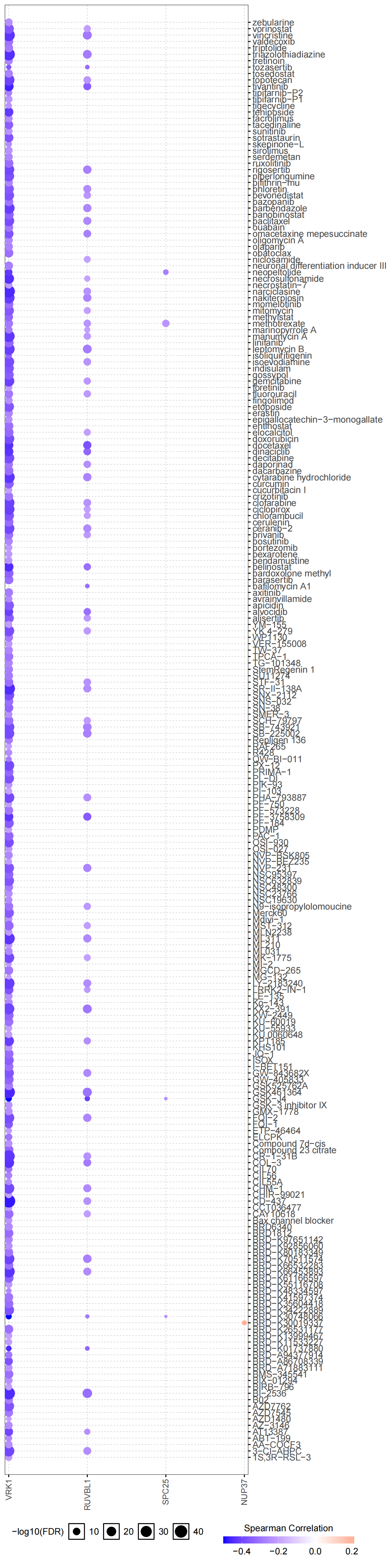

Supplement: Supplementary file 2 [file Image2.tif]

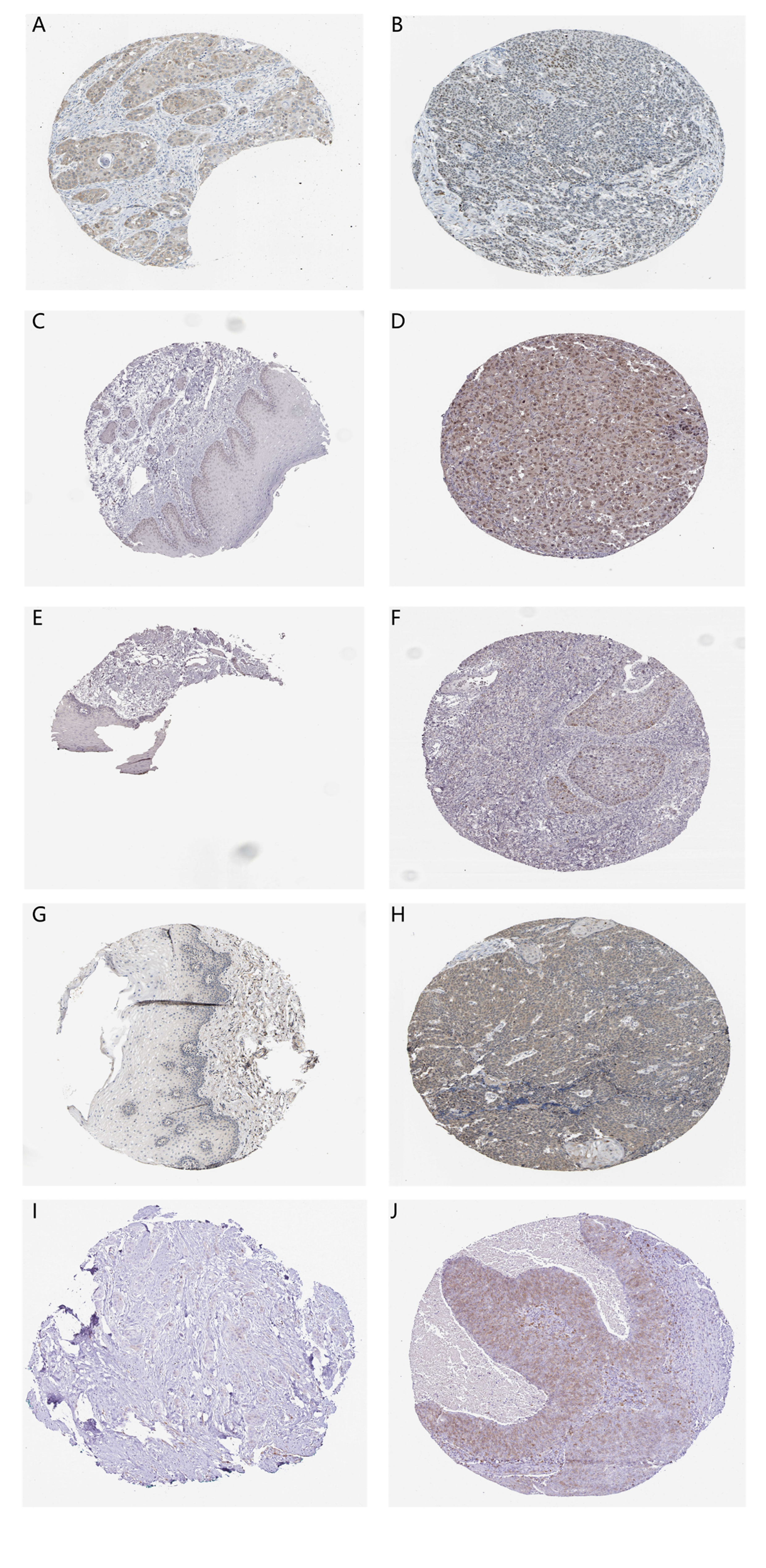

Supplement: Supplementary file 3 [file Image1.tif]
